# Subjective Outcome Evaluation of the Project P.A.T.H.S.: Findings Based on the Perspective of the Program Participants

**DOI:** 10.1100/tsw.2007.38

**Published:** 2007-01-22

**Authors:** Daniel T. L. Shek, Hing Keung Ma

**Affiliations:** ^1^ Quality of Life Centre, Hong Kong Institute of Asia-Pacific Studies, The Chinese University of Hong Kong, Shatin, Hong Kong; ^2^ Social Welfare Practice and Research Centre, Department of Social Work, The Chinese University of Hong Kong, Shatin, Hong Kong; ^3^ Department of Education Studies, Hong Kong Baptist University, Hong Kong

**Keywords:** positive youth development, adolescence, holistic health, Hong Kong, China, subjective outcome evaluation form

## Abstract

A total of 52 schools (n = 8679 students) participated in the experimental implementation phase of the project P.A.T.H.S. (Positive Adolescent Training through Holistic Social Programmes). After completion of the Tier 1 Program, students were invited to respond to the Subjective Outcome Evaluation Form (Form A) to assess their views of the program, instructors, and perceived effectiveness of the program. Based on the consolidated reports submitted by the schools to the funding body, the research team aggregated the consolidated data to form a “reconstructed” overall profile on the perceptions of the program participants. Results showed that high proportions of the respondents had positive perceptions of the program and the instructors, and roughly four-fifths of the respondents regarded the program as helpful to them. The present study provides additional support for the effectiveness of the Tier 1 Program of the P.A.T.H.S. Project in Hong Kong.

## INTRODUCTION

With the intensification of adolescent problems in the context of Hong Kong, there is a demand for adolescent positive youth development programs[1,2,3]. To promote holistic development among adolescents in Hong Kong, the Hong Kong Jockey Club Charities Trust approved HK$400 million to launch a project entitled "P.A.T.H.S. to Adulthood: A Jockey Club Youth Enhancement Scheme". The word "P.A.T.H.S." denotes Positive Adolescent Training through Holistic Social Programmes. A research team with researchers from five universities in Hong Kong was formed, with The Chinese University of Hong Kong as the lead institution and the first author as the principal investigator to develop a multiyear, universal, positive youth development program to promote holistic adolescent development in Hong Kong[4,5].

There are two tiers of programs (Tier 1 and Tier 2) in the P.A.T.H.S. Project. The Tier 1 Program is a universal, positive youth development program where students in Secondary 1 to 3 will participate in the program, normally with 20 h of training in the school year at each grade, involving 40 teaching units. In the Tier 1 Program, 15 positive youth development constructs are included in the 40 teaching units. These constructs include promotion of bonding, cultivation of resilience, promotion of social competence, promotion of emotional competence, promotion of cognitive competence, promotion of behavioral competence, promotion of moral competence, cultivation of self-determination, promotion of spirituality, development of self-efficacy, development of a clear and positive identity, promotion of beliefs in the future, provision of recognition for positive behavior, provision of opportunities for prosocial involvement, and fostering prosocial norms[3].

There are two implementation phases in this project: an experimental implementation phase and full implementation phase. For the experimental implementation phase (January 2006 to August 2006), 52 secondary schools participated in the project with the objectives of accumulating experience in program implementation and familiarizing frontline workers with the program design and philosophy. In the 2006/07 school year, the programs will be implemented on a full scale at Secondary 1 level. In 2007/08 school year, the programs will be implemented at Secondary 1 and Secondary 2 levels. In 2008/09 school year, the programs will be implemented at Secondary 1, Secondary 2, and Secondary 3 levels.

In view of the enormous financial and human resources injected in the project, evaluation of the project is of paramount importance. There are at least four evaluation questions that should be asked: (1) Are there any positive changes in the program participants (i.e., objective outcome evaluation)? (2) Are the program participants and workers satisfied with the program (i.e., subjective outcome evaluation)? (3) What happens during the program implementation process (i.e., process evaluation)? (4) What are the subjective experiences of the program participants and the workers (i.e., qualitative evaluation)? The inclusion of different evaluation mechanisms may help to triangulate the findings collected.

Several studies have documented the effectiveness of the Tier 1 Program implemented in the experimental implementation phase. First, based on four schools randomly selected from the 52 schools (n = 546 students) participating in the experimental implementation phase, objective outcome evaluation showed positive changes in the program participants in many measures of positive youth development[6]. Second, qualitative evaluation based on five focus groups (n = 43 students) recruited from four schools showed that the subjective experiences of the program participants were positive in nature[7]. Third, evaluation findings based on systematic evaluation of program implementation in six schools randomly selected from the participating schools showed that the overall level of program adherence was generally high (average adherence = 84.5%) and high implementation quality of the program in different areas (e.g., use of interactive delivery method, use of strategies to enhance student motivation, instructors' familiarity with the students) was also observed[8]. Finally, 25 schools and three school social service units were randomly selected to understand the quality of the implementation process of the Tier 1 Program of the P.A.T.H.S. Project. Results showed that nearly all workers (97.1%) regarded the program to be beneficial to the students and most of them (78.6%) had positive global evaluation of the project. In addition, while the program implementers expressed concerns about the program design and the implementation process, they generally regarded the program as helpful to the students and they had positive global evaluation of the program[9].

Although the four papers cited above described the positive program effects of the Tier 1 Program of the P.A.T.H.S. Project in the experimental implementation phase, there are two limitations involved. First, only 20 items were used to assess the participants' perceptions of the program, instructor, and the effectiveness of the program in a condensed manner at post-test. Although reliability analyses showed that the items assessing different areas were internally consistent, more items could be used to assess the perceived program effectiveness, particularly with reference to the positive youth development constructs covered in the program. Second, the subjective outcome evaluation data were collected in a sample of schools only. Ideally speaking, it would be desirable to aggregate the subjective outcome evaluation data and "reconstruct" an overall profile of the perceptions of the program participants in different schools. As the P.A.T.H.S. Project was financially supported by the Hong Kong Jockey Club Charities Trust, each participating school was required to submit an evaluation report with the consolidated subjective outcome evaluation profile of the school to the funding body. In other words, as the workers were expected to conduct program evaluation as part of their professional practice, we could make use of such reports to "reconstruct" the overall profile of the subjective outcome evaluation data. The major advantage of this strategy is that we can promote practice evaluation in the field while, at the same time, conduct secondary data analyses of the reports submitted.

## METHODS

### Participants and procedures

There were 52 schools joining the experimental implementation phase. The mean number of students per school was 166.90 (ranging from 37–240 students), with an average of 4.58 classes per school (ranging from 2–7 classes). Among them, 29 schools adopted the full program (i.e., 20-h program involving 40 units). The mean number of sessions used to implement the program was 17.75 (ranging from 3–50 sessions). While 21 (40.4%) schools incorporated the program in the formal curriculum (e.g., liberal studies, life education), 31 schools (59.6%) used other modes (e.g., using form master's periods and other combinations) to implement the program. The mean numbers of social workers and teachers implementing the program per school were 2.63 (ranging from 0–8) and 5.13 (ranging from 0–17), respectively.

After the Tier 1 Program was completed, the participants were invited to respond to a subjective outcome evaluation questionnaire. A total of 8057 students (mean = 154.94 students per school, ranging from 37–212) responded to the Subjective Outcome Evaluation Form (Form A) developed by the research team. The data collection was normally carried out at the last session of the program. On the day when the evaluation data were collected, the purpose of the evaluation was mentioned and the confidentiality of the data collected was repeatedly emphasized to all of the students. The students were asked to indicate their wish if they did not want to respond to the evaluation questionnaire (i.e., "passive" informed consent was obtained from the students). All participants responded to all scales in the evaluation form in a self-administration format. Adequate time was provided for the participants to complete the questionnaire. To facilitate the program evaluation, the research team developed an evaluation manual with standardized instructions for collecting the subjective outcome evaluation data[10]. In addition, adequate training was provided to the workers during the 20-h training workshops on how to collect and analyze the data collected by Form A.

### Instruments

The Subjective Outcome Evaluation Form (Form A) was designed by Shek and Siu[10]. Broadly speaking, there are several parts in this evaluation form as follows:

- Participants' perceptions of the program, such as program objectives, design, classroom atmosphere, interaction among the students, and the respondent's participation during class (10 items)
- Participants' perceptions of the workers, such as the preparation of the instructors, professional attitude, involvement, and interaction with the students (10 items)
- Participants' perception of the effectiveness of the program, such as promotion of different psychosocial competencies, resilience, and overall personal development (16 items)
- The extent to which the participant would recommend the program to other people with similar needs (1 item)
- The extent to which the participant would join similar programs in future (1 item)
- Overall satisfaction with the program (1 item)

- Things that the participant learned from the program (opened-ended question)
- Things that the participant appreciated most (open-ended question)
- Opinion about the instructor(s) (open-ended question)
- Areas that require improvement (open-ended question)

The workers collecting the data were requested to input the data in an Excel file developed by the research team, which would automatically compute the frequencies and percentages associated with the different ratings for an item. When the schools submitted the reports, they were also requested to submit the soft copy of the consolidated data sheets. In the report prepared by the schools, the workers were also required to estimate the degree of adherence to the program manuals (i.e., the extent to which the program is implemented in accordance with the program manuals). After receiving the consolidated data by the funding body, the data were aggregated to "reconstruct" the overall profile based on the subjective outcome evaluation data.

## RESULTS

The quantitative findings based on the closed-ended questions are presented in this paper. There are several observations that can be highlighted from the findings. First, roughly three-quarters of the respondents perceived the program in a positive manner (Table 1). For example, 79% of the students indicated that they participated actively during class; 75% felt that the classroom atmosphere was pleasant. Second, a high proportion of the students had positive evaluation of the instructors (Table 2). For example, 86% of the respondents indicated that the instructor was ready to provide help to them when needed; 87% of the respondents perceived that the instructor encouraged the students. Third, as shown in Table 3, roughly four-fifths of the respondents perceived that the program promoted their development, including social competence (81%), emotional competence (80%), and overall development (81%). Fourth, while roughly three-quarters of the participants would recommend the program to their friends when they have similar needs, only a simple majority of them (63.18%) would join similar programs in future (Table 4). Finally, roughly four-fifths of the respondents indicated that they were satisfied with the program (Table 4). Regarding the degree of program adherence estimated by the workers, the mean level of adherence was 85.44%, with a range from 50–100%.

## DISCUSSION

Subjective outcome evaluations or client satisfaction surveys are commonly used to assess the perceived benefits of a program and the degree of satisfaction that the participants have regarding the different aspects of the program. The use of subjective outcome indicators or the client satisfaction approach in evaluation has a long history in human services in different cultures[11,12,13,14]. Although there are arguments against the use of subjective outcome assessments, there is evidence showing that subjective outcome measures were correlated with objective outcome measures[15,16,17,18,19,20,21].

The findings based on the subjective outcome evaluation strategy or client satisfaction survey showed that a high proportion of the respondents had positive perceptions of the program and the worker. Most importantly, roughly four-fifths of the respondents regarded the program as helpful to them. In short, the subjective outcome evaluation findings generally showed that the program participants had a positive perception of the program as well as the workers who implemented the program. Nevertheless, it is noteworthy that although most of the participants had positive perceptions of the program, workers, and perceived effectiveness of the program, and roughly three-quarters of the participants indicated that they would recommend the program to peers with similar needs, only 63% of them indicated that they would participate in similar programs in future. With further analyses of the qualitative data collected, answers to this puzzle may be generated.

In the limited Western studies on the quality of program implementation, studies generally showed that the degree of program adherence was not high. For example, Ringwalt et al.[22] found that one-fifth of the workers implementing the program did not use the curriculum guide at all and only 15% of them followed very closely. In contrast to the Western findings, the estimated level of program adherence was quite high in the present study. Actually, the mean estimated degree of adherence in the present study (85.44%) was highly comparable to the figure (84.50%) based on the ratings made by trained observers in a random sample of six schools. It is conjectured that the training provided to the workers as well as the commitment of the workers (as reflected by the perceptions of the students) contributed to this high level of program adherence.

**TABLE 1**
**Summary of the Views of the Program Participants**

| | Strongly Disagree (1) | | Disagree (2) | | Slightly Disagree (3) | | Slightly Agree (4) | | Agree (5) | | Strongly Agree (6) | | Participants with Positive Response (Options 4–6) | |
|---|---|---|---|---|---|---|---|---|---|---|---|---|---|---|
| | N | % | N | % | N | % | N | % | N | % | N | % | N | % |
| The objectives of the curriculum are very clear (n = 8030). | 311 | 3.87 | 330 | 4.11 | 952 | 11.86 | 2674 | 33.30 | 2922 | 36.39 | 841 | 10.47 | 6437 | 80.16 |
| The design of the curriculum is very good (n = 8027). | 318 | 3.96 | 448 | 5.58 | 1199 | 14.94 | 2888 | 35.98 | 2486 | 30.97 | 688 | 8.57 | 6062 | 75.52 |
| The activities were carefully planned (n = 8014). | 294 | 3.67 | 425 | 5.30 | 1103 | 13.76 | 2833 | 35.35 | 2655 | 33.13 | 704 | 8.78 | 6192 | 77.26 |
| The classroom atmosphere was very pleasant (n = 7996). | 364 | 4.55 | 486 | 6.08 | 1120 | 14.01 | 2381 | 29.78 | 2511 | 31.40 | 1134 | 14.18 | 6026 | 75.36 |
| There was much peer interaction among the students (n = 7949). | 314 | 3.95 | 385 | 4.84 | 970 | 12.20 | 2390 | 30.07 | 2669 | 33.58 | 1221 | 15.36 | 6280 | 79.00 |
| I participated actively during lessons (including discussions, sharing, games, etc.) (n = 8005). | 326 | 4.07 | 373 | 4.66 | 957 | 11.96 | 2621 | 32.74 | 2555 | 31.92 | 1173 | 14.65 | 6349 | 79.31 |
| I was encouraged to do my best (n = 8017). | 368 | 4.59 | 473 | 5.90 | 1219 | 15.21 | 2766 | 34.50 | 2400 | 29.94 | 791 | 9.87 | 5957 | 74.30 |
| The learning experience I encountered enhanced my interest towards the lessons (n = 7986). | 421 | 5.27 | 503 | 6.30 | 1248 | 15.63 | 2681 | 33.57 | 2328 | 29.15 | 805 | 10.08 | 5814 | 72.80 |
| Overall, I have very positive evaluation of the program (n = 7980). | 422 | 5.29 | 560 | 7.02 | 1215 | 15.23 | 2698 | 33.81 | 2328 | 29.17 | 757 | 9.49 | 5783 | 72.47 |
| On the whole, I like this curriculum very much (n = 7990). | 542 | 6.78 | 548 | 6.86 | 1084 | 13.57 | 2459 | 30.78 | 2307 | 28.87 | 1050 | 13.14 | 5816 | 72.79 |

There are three strengths of this study. First, the subjective outcome evaluation findings are based on a large sample size (n = 8679 students involving 52 schools). Such a big sample size substantially enhances the generalizability of the research findings to other student populations. Second, different aspects of subjective outcome, including views of the program, worker, perceived effectiveness, and overall satisfaction were covered in the study. Third, the present study demonstrates the strategy of “reconstructing” the overall profile of the subjective outcomes based on the reports submitted by the participating schools. In fact, this study is the first published scientific study utilizing this "reconstruction" approach based on such a large number of students in the Chinese culture.

**TABLE 2**
**Summary of the Views of the Program Participants of the Workers Implementing the Program**

| | Strongly Disagree (1) | | Disagree (2) | | Slightly Disagree (3) | | Slightly Agree (4) | | Agree (5) | | Strongly Agree (6) | | Participants with Positive Response (Options 4–6) | |
|---|---|---|---|---|---|---|---|---|---|---|---|---|---|---|
| | N | % | N | % | N | % | N | % | N | % | N | % | N | % |
| The instructor(s) had a good mastery of the curriculum (n = 8029). | 280 | 3.49 | 264 | 3.29 | 767 | 9.55 | 2523 | 31.42 | 2918 | 36.34 | 1277 | 15.90 | 6718 | 83.67 |
| The instructor(s) was well prepared for the lessons (n = 8020). | 237 | 2.96 | 253 | 3.15 | 611 | 7.62 | 2308 | 28.78 | 3098 | 38.63 | 1513 | 18.87 | 6919 | 86.27 |
| The instructor(s)' teaching stills were good (n = 8002). | 264 | 3.30 | 269 | 3.36 | 786 | 9.82 | 2369 | 29.61 | 3055 | 38.18 | 1259 | 15.73 | 6683 | 83.52 |
| The instructor(s) showed good professional attitudes (n = 8013). | 254 | 3.17 | 241 | 3.01 | 695 | 8.67 | 2283 | 28.49 | 3079 | 38.43 | 1461 | 18.23 | 6823 | 85.15 |
| The instructor(s) was very involved (n = 8011). | 247 | 3.08 | 210 | 2.62 | 593 | 7.40 | 2130 | 26.59 | 3105 | 38.76 | 1726 | 21.55 | 6961 | 86.89 |
| The instructor(s) encouraged students to participate in the activities (n = 7992). | 267 | 3.34 | 207 | 2.59 | 572 | 7.16 | 2173 | 27.19 | 3075 | 38.48 | 1698 | 21.25 | 6946 | 86.91 |
| The instructor(s) cared for the students (n = 8000). | 294 | 3.68 | 248 | 3.10 | 714 | 8.93 | 2331 | 29.14 | 2904 | 36.30 | 1509 | 18.86 | 6744 | 84.30 |
| The instructor(s) was ready to offer help to students when needed (n = 7997). | 250 | 3.13 | 232 | 2.90 | 623 | 7.79 | 2284 | 28.56 | 3044 | 38.06 | 1564 | 19.56 | 6892 | 86.18 |
| The instructor(s) had much interaction with the students (n = 8006). | 292 | 3.65 | 332 | 4.15 | 848 | 10.59 | 2416 | 30.18 | 2820 | 35.22 | 1298 | 16.21 | 6534 | 81.61 |
| Overall, I have very positive evaluation of the instructors (n = 8018). | 294 | 3.67 | 232 | 2.89 | 617 | 7.70 | 2095 | 26.13 | 3081 | 38.43 | 1699 | 21.19 | 6875 | 85.74 |

Of course, several limitations of the present study should be highlighted. First, as the data were reconstructed from the reports submitted by the schools, the unit of analysis was the schools rather than the individual program participants. While this limitation did not affect the reconstruction of the percentage and rating data, this will adversely affect future analyses involving relationships among variables (e.g., relationship between perception of the program and perceived program effectiveness) because only 52 rather than 8679 pairs of data points were involved. This would substantially reduce the power of statistical analyses. Second, while the present findings are interpreted in terms of the positive program effects and experiences of the program participants, it should be noted that there are several alternative explanations. The first alternative explanation is that the students were afraid that they would be punished by the workers if they did not respond in the favorable direction. Actually, this alternative explanation can be partially dismissed because the students responded in an anonymous manner. The second alternative explanation is that the students consciously acted in a "nice" manner to help the workers to illustrate positive program effect. However, this alternative explanation could be partially dismissed because negative ratings were recorded (e.g., whether the participants would join the program again) and the students responded in an anonymous manner. The third alternative explanation is that the high proportions of positive responses observed, in fact, were random responses (i.e., the students did not respond in a serious manner). However, this alternative explanation can also be dismissed because reliability analyses showed that the whole scale was internally consistent. Despite these limitations, the

**TABLE 3**
**Perceived Effectiveness of the Program by the Program Participants**

| The extent to which the course (i.e., the program that all students have joined) has helped you (n = No. of responses) | Unhelpful (1) | | Not Very Helpful (2) | | Slightly Helpful (3) | | Helpful (4) | | Very Helpful (5) | | Participants with Positive Response (Options 3–5) | |
|---|---|---|---|---|---|---|---|---|---|---|---|---|
| | N | % | N | % | N | % | N | % | N | % | N | % |
| It has strengthened my bonding with teachers, classmates, and family (n = 8034). | 607 | 7.56 | 1392 | 17.33 | 3220 | 40.08 | 2158 | 26.86 | 657 | 8.18 | 6035 | 75.12 |
| It has strengthened my resilience in adverse conditions (n = 8035). | 457 | 5.69 | 1275 | 15.87 | 2963 | 36.88 | 2532 | 31.51 | 808 | 10.06 | 6303 | 78.44 |
| It has enhanced my social competence (n = 8019). | 428 | 5.34 | 1082 | 13.49 | 2898 | 36.14 | 2594 | 32.35 | 1017 | 12.68 | 6509 | 81.17 |
| It has improved my ability to handle and express my emotions (n = 8022). | 489 | 6.10 | 1090 | 13.59 | 2812 | 35.05 | 2630 | 32.78 | 1001 | 12.48 | 6443 | 80.32 |
| It has enhanced my cognitive competence (n = 8026). | 456 | 5.68 | 1168 | 14.55 | 2927 | 36.47 | 2483 | 30.94 | 992 | 12.36 | 6402 | 79.77 |
| My ability to resist harmful influences has been improved (n = 8018). | 474 | 5.91 | 1114 | 13.89 | 2761 | 34.44 | 2594 | 32.35 | 1075 | 13.41 | 6430 | 80.19 |
| It has strengthened my ability to distinguish between the good and the bad (n = 8027). | 411 | 5.12 | 970 | 12.08 | 2859 | 35.62 | 2642 | 32.91 | 1145 | 14.26 | 6646 | 82.80 |
| It has increased my competence in making sensible and wise choices (n = 8020). | 414 | 5.16 | 1072 | 13.37 | 2804 | 34.96 | 2648 | 33.02 | 1082 | 13.49 | 6534 | 81.47 |
| It has helped me to have life reflections (n = 8026). | 593 | 7.39 | 1084 | 13.51 | 2697 | 33.60 | 2488 | 31.00 | 1164 | 14.50 | 6349 | 79.11 |
| It has reinforced my self-confidence (n = 8019). | 545 | 6.80 | 1209 | 15.08 | 2712 | 33.82 | 2429 | 30.29 | 1124 | 14.02 | 6265 | 78.13 |
| It has increased my self-awareness (n = 8020). | 510 | 6.36 | 1111 | 13.85 | 2804 | 34.96 | 2496 | 31.12 | 1099 | 13.70 | 6399 | 79.79 |
| It has helped me to face the future with a positive attitude (n = 8006). | 520 | 6.50 | 1129 | 14.10 | 2717 | 33.94 | 2536 | 31.68 | 1104 | 13.79 | 6357 | 79.40 |
| It has helped me to cultivate compassion and care about others (n = 8016). | 489 | 6.10 | 1055 | 13.16 | 2828 | 35.28 | 2503 | 31.23 | 1141 | 14.23 | 6472 | 80.74 |
| It has encouraged me to care about the community (n = 8013). | 554 | 6.91 | 1215 | 15.16 | 2773 | 34.61 | 2396 | 29.90 | 1075 | 13.42 | 6244 | 77.92 |
| It has promoted my sense of responsibility in serving the society (n = 8020). | 511 | 6.37 | 1170 | 14.59 | 2816 | 35.11 | 2441 | 30.44 | 1082 | 13.49 | 6339 | 79.04 |
| It has enriched my overall development (n = 8016). | 495 | 6.18 | 1032 | 12.87 | 2550 | 31.81 | 2494 | 31.11 | 1445 | 18.03 | 6489 | 80.95 |

present findings suggest that the Tier 1 Program and its implementation were perceived in a positive manner by the program participants and they perceived the program to be beneficial to their own development. These positive findings are important because they suggest that the program can successfully engage the students in the program implementation process. From a program evaluation point of view, as systematic evaluation of social services is at its infancy in different Chinese contexts, the present paper constitutes a model based on which future subjective outcome evaluation studies can be conducted[23,24].

**TABLE 4**
**Other Aspects of Subjective Outcome Evaluation**

**If your friends have needs and conditions similar to yours, will you suggest him/her to join this course? (n = 7935)**

| Definitely Will Not Suggest (1) | | Will Not Suggest (2) | | Will Suggest (3) | | Definitely Will Suggest (4) | | Participants with Positive Responses (Options 3 and 4) | |
|---|---|---|---|---|---|---|---|---|---|
| N | % | N | % | N | % | N | % | N | % |
| 641 | 8.08 | 1237 | 15.59 | 4732 | 59.63 | 1325 | 16.70 | 6057 | 76.33 |

**Will you participate in similar courses again in the future? (n = 7938)**

| Definitely Will Not Participate (1) | | Will Not Participate (2) | | Will Participate (3) | | Definitely Will Participate (4) | | Participants with Positive Responses (Options 3 and 4) | |
|---|---|---|---|---|---|---|---|---|---|
| N | % | N | % | N | % | N | % | N | % |
| 1005 | 12.66 | 1918 | 24.16 | 3857 | 48.59 | 1158 | 14.59 | 5015 | 63.18 |

**On the whole, are you satisfied with this course? (n = 7935)**

| Very Dissatisfied (1) | | Moderately Dissatisfied (2) | | Slightly Dissatisfied (3) | | Satisfied (4) | | Moderately Satisfied (5) | | Very Satisfied (6) | | Participants with Positive Responses (Options 4–6) | |
|---|---|---|---|---|---|---|---|---|---|---|---|---|---|
| N | % | N | % | N | % | N | % | N | % | N | % | N | % |
| 407 | 5.13 | 421 | 5.31 | 716 | 9.02 | 3295 | 41.52 | 2118 | 26.69 | 978 | 12.33 | 6391 | 80.54 |

## ACKNOWLEDGMENTS

The preparation for this paper and the Project P.A.T.H.S. were financially supported by the Hong Kong Jockey Club Charities Trust.
